# *Aliikangiella litoralis* sp. nov. and *Aliikangiella aequoris* sp. nov., isolated from coastal seawaters of the Yellow Sea

**DOI:** 10.71150/jm.2602008

**Published:** 2026-05-19

**Authors:** Seungyeop Oh, Yeonjung Lim, Meora Rajeev, Jang-Cheon Cho

**Affiliations:** 1 Department of Biological Sciences and Bioengineering, Inha University, Incheon 22212, Republic of Korea; 2 Center for Molecular and Cell Biology, Inha University, Incheon 22212, Republic of Korea; 3 Institute for Specialized Teaching and Research, Inha University, Incheon 22212, Republic of Korea

**Keywords:** *Aliikangiella litoralis*, *Aliikangiella aequoris*, polyphasic taxonomy, marine bacteria, novel species, genome

## Abstract

Three Gram-stain-negative, strictly aerobic, motile bacterial strains, designated IMCC44359^T^, IMCC44632^T^, and IMCC44653, were isolated from coastal surface seawater collected near Jangbong Island in the Yellow Sea. Phylogenetic analyses based on 16S rRNA gene and whole-genome sequences assigned the isolates to the genus *Aliikangiella*. Strains IMCC44632^T^ and IMCC44653 shared identical 16S rRNA gene sequences and exhibited high genomic relatedness (99.0% average nucleotide identity and 92.0% digital DNA-DNA hybridization), indicating that they represent a single species. In contrast, strain IMCC44359^T^ showed low genomic relatedness to these strains and to previously validly published *Aliikangiella* species, supporting its recognition as a distinct species. The genome of IMCC44359^T^ (5.95 Mbp; 36.5 mol% G + C) is substantially larger than those of IMCC44632^T^ and IMCC44653 (~3.75 Mbp; 40.1–40.2 mol% G + C), and all genomes encode aerobic chemoorganotrophic metabolism and biochemical capacities consistent with adaptation to marine environments. The isolates grew under mesophilic and moderately halophilic conditions typical of coastal seawater bacteria, with growth occurring at ranges at 10–40℃, pH 6.0–9.0, and 0.5–7.5% NaCl (optimum, 30℃, pH 7.0–8.0, and 2.0–3.0% NaCl). All strains contained ubiquinone-8 (Q-8) as the sole respiratory quinone, and phosphatidylethanolamine, phosphatidylglycerol, and diphosphatidylglycerol were the major polar lipids. The dominant cellular fatty acids were iso-C_15:0_ and summed feature 9 (iso-C_17:1_ ω9c and/or C_16:0_ 10-methyl). Integrated phylogenetic, genomic, phenotypic evidence supported the recognition of two novel species within the genus *Aliikangiella*, for which the names *Aliikangiella litoralis* sp. nov. (type strain IMCC44359^T^ = KCTC 18089^T^ = JCM 37879^T^ = HNIBRBA19635^T^) and *Aliikangiella aequoris* sp. nov. (type strain IMCC44632^T^ = KCTC 18090^T^ = JCM 37880^T^ = HNIBRBA19636^T^) are proposed.

## Introduction

The family *Kangiellaceae* currently includes three genera, *Kangiella*, *Pleionea*, and *Aliikangiella*. The type genus *Kangiella* (Yoon et al., 2004) encompasses 11 validly published species that are non-motile rods, whereas *Pleionea* and *Aliikangiella* include both non-motile and flagellated taxa, indicating phenotypic diversification within the family. Although Wang et al. (2020) proposed the family *Pleioneaceae* to accommodate *Pleionea* and *Aliikangiella*, this family is treated as a heterotypic synonym of *Kangiellaceae* in the LPSN (List of Prokaryotic names with Standing in Nomenclature) framework (Parte et al., 2020). Overall, members of these genera share broadly conserved chemotaxonomic features yet differ in motility and genome characteristics, consistent with diversification within the family *Kangiellaceae*.

The genus *Aliikangiella* (family *Kangiellaceae*, order *Oceanospirillales*) was established by Wang et al. (2015) with *Aliikangiella marina* as the type species, which was isolated from a culture of the marine microalga *Picochlorum* sp. The taxonomic scope of the genus was later expanded by the description of *Aliikangiella coralliicola* from coral-associated samples (Wang et al., 2020). At present, the genus comprises two validly published species and one not-yet-validly published species, “*Aliikangiella maris*” (Li et al., 2025), which was isolated from the marine alga *Phaeocystis globosa*. Notably, *Aliikangiella* has so far been recovered only from marine settings (e.g., microalgal culture systems and coral-associated habitats), implying adaptation to marine-associated ecological niches.

Species of *Aliikangiella* are typically mesophilic and moderately halophilic, with growth optima compatible with marine conditions. Reported chemotaxonomic traits include ubiquinone-8 (Q-8) as the major respiratory quinone and phosphatidylethanolamine (PE), phosphatidylglycerol (PG), and diphosphatidylglycerol (DPG) as major polar lipids. Comparative genomics has also suggested that *Aliikangiella* possesses relatively large genomes (5.34–7.04 Mbp) compared with other *Kangiellaceae* genera such as *Kangiella* (2.46–2.85 Mbp) and *Pleionea* (4.04–5.19 Mbp), potentially reflecting broader functional capacity.

Island-associated coastal waters around the Korean Peninsula are biologically dynamic and have repeatedly yielded novel bacterial taxa (Han et al., 2025; Oh et al., 2026; Tak et al., 2024; Yang et al., 2024). During ongoing cultivation-based surveys of coastal microbial diversity, strains IMCC44359^T^, IMCC44632^T^, and IMCC44653 were isolated from surface seawater collected near Jangbong Island in the Yellow Sea. A polyphasic taxonomic assessment indicated that these isolates represent two previously unrecognized novel species within the genus *Aliikangiella*. In the present study, we report their phylogenetic, genomic, and phenotypic characteristics and propose *Aliikangiella litoralis* sp. nov. and *Aliikangiella aequoris* sp. nov.

## Materials and Methods

### Isolation and culture conditions

Strain IMCC44359^T^ was isolated from coastal surface seawater collected at Jangbong Island, Incheon, Republic of Korea (37.55172° N, 126.33675° E) in June 2023. Strains IMCC44632^T^ and IMCC44653 were obtained from seawater collected at a different coastal beach on Jangbong Island (37.54072° N, 126.32931° E) during the same period. Serially diluted seawater samples were spread on marine agar 2216 (MA; BD Difco, USA). After incubation at 20°C for 5 days, colonies were purified by three successive transfers. The isolates were routinely cultivated on MA at 30°C and preserved at −80°C as glycerol stocks (10%, v/v). For comparative phenotypic analyses, *A. marina* KCTC 42667^T^ (= GYP-15^T^) and *A. coralliicola* KCTC 72442^T^ (= M105^T^) were obtained from the Korean Collection for Type Cultures (KCTC) and grown on MA at 30°C for 5 day; meanwhile, the phenotypic characteristics of “*A. maris*” GXAS 306^T^ were obtained from the published literature (Li et al., 2025), having been generated under growth conditions identical or comparable to those used in this study (e.g., MA at 30°C).

### 16S rRNA gene-based phylogenetic analysis

The 16S rRNA gene sequences of strains IMCC44359^T^, IMCC44632^T^, and IMCC44653 were obtained by PCR from cell lysates prepared in Tris–EDTA buffer using mechanical disruption with glass beads and repeated freeze–thaw cycles. Amplification was performed with universal bacterial primers 27F and 1492R (Weisburg et al., 1991). Sanger sequencing (Macrogen Inc., Korea) used primers 27F, 518F, 800R, and 1492R. Sequence comparisons were conducted using BLASTn against GenBank, and pairwise similarities were calculated with EzBioCloud (Chalita et al., 2024).

For phylogenetic reconstruction, sequences were aligned with the SINA aligner (SILVA Incremental Aligner; v1.2.12) (Pruesse et al., 2012) and curated in ARB (Ludwig et al., 2004). Tree inference was performed in MEGA X (Kumar et al., 2018) using maximum-likelihood (ML) (Felsenstein, 1981), neighbor-joining (NJ) (Saitou and Nei, 1987), and minimum-evolution (ME) (Rzhetsky and Nei, 1992) algorithms. For ML analysis, the best-fit nucleotide substitution model was determined in MEGA X based on the Bayesian Information Criterion (BIC), and the ML tree was reconstructed under the selected model. NJ and ME trees were generated using evolutionary distances calculated with the Kimura two-parameter model (Kimura, 1980). The robustness of tree topologies was evaluated by bootstrap analysis with 1,000 replicates (Felsenstein, 1985).

### Whole-genome sequencing and analysis

Approximately 50 mg (wet weight) of biomass from MA plates was resuspended in DNA/RNA Shield (Zymo Research, USA) and shipped to MicrobesNG (UK). Whole-genome sequencing was performed using a hybrid approach that combined Illumina short-read and Oxford Nanopore long-read platforms. Illumina libraries were prepared with the Nextera XT DNA Library Preparation Kit and sequenced on a NextSeq 2000 platform to generate 250-bp paired-end reads. Nanopore libraries were prepared with the SQK-RBK114.96 kit and sequenced on a PromethION system with a FLO-PRO114M flow cell. Hybrid assemblies were produced using Hybracter v0.11.2 (Bouras et al., 2024). Genome completeness and contamination were evaluated with CheckM2 v1.1.0 (Chklovski et al., 2023).

Genome-based taxonomic analyses were conducted following the framework proposed by Riesco and Trujillo (2024). Species-level relatedness was assessed by calculating average nucleotide identity (ANI) values using JSpeciesWS (Richter et al., 2016) and by estimating digital DNA–DNA hybridization (dDDH) values with the Genome-to-Genome Distance Calculator (GGDC 3.0) (Meier-Kolthoff et al., 2013). For genus-level comparisons, average amino acid identity (AAI) values were calculated using EzAAI v1.2.3 (Kim et al., 2021), and the percentage of conserved proteins (POCP) was determined using the Nextflow-based POCP pipeline (hoelzer/pocp, v2.3.6) following Hölzer (2024), with protein alignments performed using DIAMOND. Reference genome sequences for comparative analyses were retrieved from the NCBI Datasets database (O’Leary et al., 2024).

Genome-based phylogenetic relationships were inferred using GTDB-Tk v2.4.0 (Chaumeil et al., 2022) with the bacterial bac120 marker gene set from the Genome Taxonomy Database (GTDB; release R226). The concatenated alignment of 120 single-copy marker genes was analyzed under the maximum-likelihood framework using RAxML v8.2.12 (Stamatakis, 2014). Functional genome annotation was performed with Prokka v1.14.6 (Seemann, 2014). Predicted protein sequences were assigned to KEGG orthologs using BlastKOALA (Kanehisa et al., 2016), and metabolic pathway completeness was assessed using the kegg-pathway-completeness tool v1.3.0 (https://github.com/EBI-Metagenomics/kegg-pathways-completeness-tool).

### Physiological and biochemical characterization

Cell morphology was examined by transmission electron microscopy (CM200, Philips, Netherlands) after negative staining. Cells were suspended in 0.2 M cacodylate buffer, stained with 1% (w/v) uranyl acetate (Electron Microscopy Sciences, USA), and mounted on Formvar-coated copper grids (FCF300-CU; Ted Pella, USA). The Gram reaction was assessed using the KOH-based non-staining method (Powers, 1995). Catalase activity was tested with 3% (v/v) H_2_O_2_, and oxidase activity was determined using 1% (w/v) Kovac’s reagent (bioMérieux, France). Motility was assessed in soft MA (0.5%, BD Difco, USA) by observing outward diffusion from the inoculation point. Temperature-dependent growth was examined on MA at 4°C and at 10–50°C (5°C intervals). NaCl tolerance was tested on NaCl-free MA supplemented to final concentrations of 0–4.0% (w/v; 0.5% increments), 5%, 7.5%, 10%, 15%, and 20%. pH range and optimum were assessed in marine broth (MB; BD Difco, USA) adjusted to pH 3.5–10.5 (0.5-unit increments) using citrate, MES, MOPS, HEPES, Tris, and CHES buffers (Sigma-Aldrich, USA), with growth monitored by OD_600_ (UV2600; Shimadzu, Japan). Anaerobic growth was examined using the GasPak^TM^ EZ Anaerobe Pouch System with Indicator (BD Diagnostics, USA). To verify anaerobic conditions, the anaerobic bacterium *Ferrimonas sediminicola* IMCC35001^T^ was inoculated as a positive control. Hydrolytic activities were tested on MA supplemented with starch (1%, w/v), carboxymethyl cellulose (1%, w/v), chitin (1%, w/v), casein (3% skim milk, w/v), Tween 20 (1%, v/v), and or Tween 80 (1%, v/v) (Sigma-Aldrich, USA). DNA degradation was assessed using DNase Test Agar (BD Diagnostics, USA), and H_2_S production was tested on Triple Sugar Iron (TSI) agar (BD Difco, USA). Esculin hydrolysis activity was determined using Esculin Agar (MB-E2166, MB Cell). Additional biochemical characteristics were determined using API 20NE, API ZYM kits (bioMérieux, France), and the GEN III MicroPlate (Biolog, USA) with salinity adjusted to 2% NaCl.

### Chemotaxonomic characterization

Fatty acid methyl ester (FAME) analysis was performed using biomass from IMCC44359^T^, IMCC44632^T^, IMCC44653, *A. marina* KCTC 42667^T^, and *A. coralliicola* KCTC 72442^T^ grown on MA at 30°C for 5 days. Fatty acids were analyzed using an Agilent 7890 GC (Agilent Technologies, USA) with the Sherlock MIS (MIDI, USA) v6.1 and TSBA6 database (Sasser, 1990). Polar lipids were extracted as described by Minnikin et al. (1984) and separated by two-dimensional TLC on silica gel 60 F254 plates (Merck, Germany). Solvent systems were chloroform/methanol/water (60:30:4, v/v) in the first dimension and chloroform/acetic acid/methanol/water (40:7.5:6:1.8, v/v) in the second. Total lipids were visualized with molybdophosphoric acid, and lipid classes were detected using ninhydrin (aminolipids), molybdenum blue (phospholipids), α-naphthol (glycolipids), and Dragendorff’s reagent (phosphatidylcholine). Respiratory quinones were co-extracted and analyzed by reverse-phase HPTLC on RP-18 F254 plates (Merck, Germany) following Collins and Jones (1981).

### Nucleotide sequence accession numbers

The 16S rRNA gene sequences of strains IMCC44359^T^, IMCC44632^T^, and IMCC44653 have been deposited in the GenBank/EMBL/DDBJ databases under accession numbers PX239437, PX239438, and PX239439, respectively. The complete genome sequences are available under accession numbers CP199292 (IMCC44359^T^), JBSKFV000000000 (IMCC44632^T^), and JBSKFW000000000 (IMCC44653).

## Results and Discussion

### 16S rRNA gene-based phylogeny

Nearly full-length 16S rRNA gene sequences were obtained for IMCC44359^T^ (1,464 bp), IMCC44632^T^ (1,441 bp), and IMCC44653 (1,437 bp). Strains IMCC44359^T^ and IMCC44632^T^ shared 97.2% sequence similarity, which is below the commonly accepted 98.7% threshold for species delineation based on 16S rRNA gene similarity (Riesco and Trujillo, 2024), indicating that they represent distinct species-level lineages. In contrast, strains IMCC44632^T^ and IMCC44653 exhibited identical 16S rRNA gene sequences (100% similarity), indicating that they may belong to the same species. Accordingly, genome-based relatedness analyses were performed to further clarify species delineation.

**Fig. 1.**
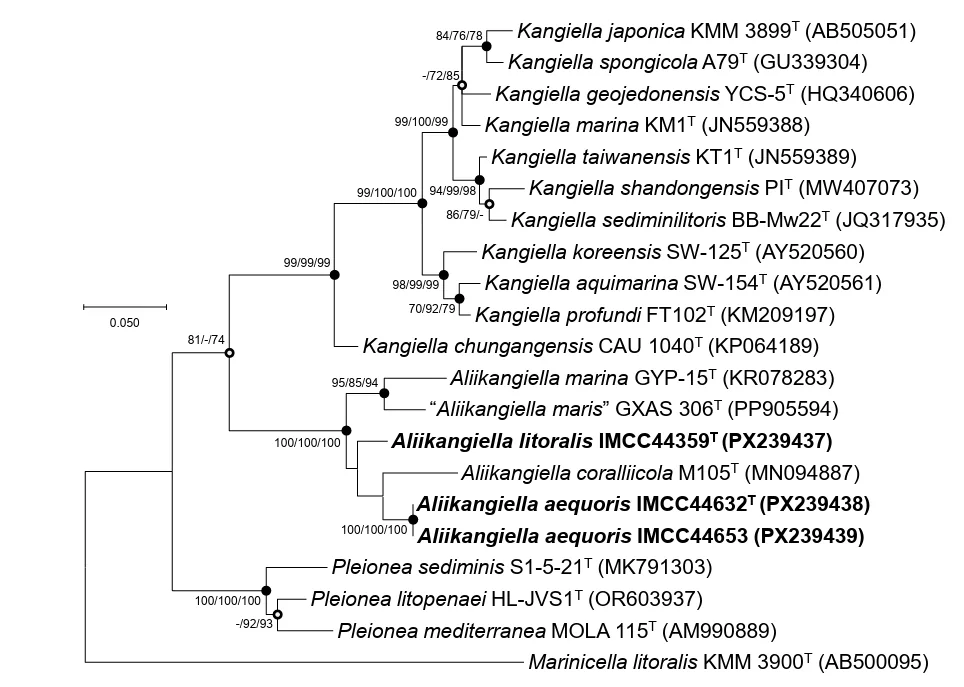
Maximum-likelihood phylogenetic tree based on 16S rRNA gene sequences showing the phylogenetic placement of strains IMCC44359^T^, IMCC44632^T^, and IMCC44653. Bootstrap support values (≥ 70%) from the maximum-likelihood, neighbor-joining, and minimum-evolution methods (in that order) are shown at the nodes. Filled circles indicate nodes recovered by all three tree-building methods, whereas open circles denote nodes supported by two of the three methods. Scale bar, substitutions per nucleotide position.

BLASTn/EzBioCloud comparisons and phylogenetic reconstructions supported assignment of the three strains to the genus *Aliikangiella*. Strain IMCC44359^T^ exhibited the highest sequence similarity to “*A. maris*” GXAS 306^T^ (96.0%), followed by *A. marina* GYP-15^T^ (95.3%) and *A. coralliicola* M105^T^ (95.3%). Both strains IMCC44632^T^ and IMCC44653 exhibited 96.4%, 94.9%, and 94.5% similarity to *A. coralliicola* M105^T^, “*A. maris*” GXAS 306^T^, and *A. marina* GYP-15^T^, respectively. All similarity values were well below 98.7%, indicating that the IMCC isolates represent phylogenetically distinct lineages from previously described *Aliikangiella* species. In the 16S rRNA gene tree, strains IMCC44359^T^, IMCC44632^T^, and IMCC44653 clustered within the family *Kangiellaceae* and formed a distinct, strongly supported clade within the genus *Aliikangiella*, positioned close to the genus *Pleionea* (Fig. 1).

### Genome characteristics and phylogenomic analysis

**Fig. 2.**
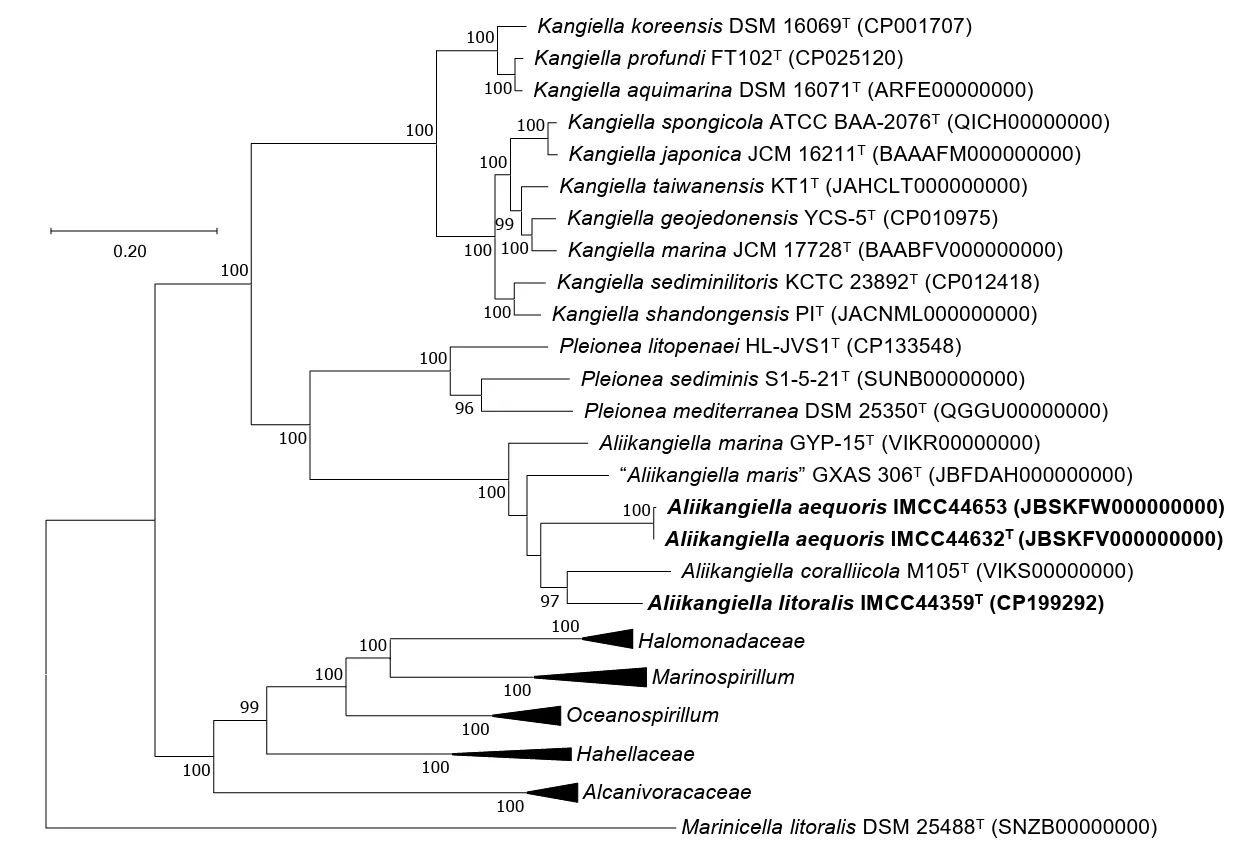
Maximum-likelihood phylogenomic tree showing the relationships of strains IMCC44359^T^, IMCC44632^T^, and IMCC44653 to related type strains. The tree was reconstructed using GTDB-Tk (bac120 marker set) and RAxML based on concatenated amino acid sequences of 120 conserved single-copy marker genes. Bootstrap support values (≥ 70%) are shown at the nodes. Scale bar, substitutions per amino acid position.

Each genome was assembled into a single circular chromosome. Genome sizes were 5,951,045 bp (IMCC44359^T^), 3,753,390 bp (IMCC44632^T^), and 3,750,766 bp (IMCC44653), with DNA G + C contents of 36.5%, 40.1%, and 40.2%, respectively. CheckM2 estimates indicated high completeness (100% for all) with low contamination (1.7% for IMCC44359^T^; < 0.5% for IMCC44632^T^ and IMCC44653). Genome annotation predicted 4,928 protein-coding genes for IMCC44359^T^ and 3,229/3,231 for IMCC44632^T^/IMCC44653. Strain IMCC44359^T^ contained 50 tRNA genes and six rRNA genes, whereas IMCC44632^T^ and IMCC44653 each harbored 43 tRNA genes and nine rRNA genes. The substantial genome size difference between strain IMCC44359^T^ (~5.95 Mb) and strains IMCC44632^T^ and IMCC44653 (~3.75 Mb) is unlikely to be attributable to assembly artifacts or contamination, given the high completeness and low contamination of the genome assemblies. This difference likely reflects variation in gene content and functional potential between the *Aliikangiella* species, consistent with their genomic distinctiveness. Comparative genome features for the three strains and three *Aliikangiella* species are summarized in Table S1. Overall genome relatedness indices clearly supported the presence of two species among the isolates. Strains IMCC44632^T^ and IMCC44653 exhibited ANI and dDDH values of 99.0% and 92.0%, respectively, consistent with a single genomic species. In contrast, comparisons between IMCC44359^T^ and IMCC44632^T^/IMCC44653 yielded ANI and dDDH values of 69.9% and 18.6%, respectively. Relative to the three *Aliikangiella* species, all three strains showed low genomic relatedness values (ANI 68.6–71.3%; dDDH 18.0–20.7%). Using the accepted thresholds of 95–96% ANI and 70% dDDH (Riesco and Trujillo, 2024), these data indicate that IMCC44359^T^ represents one novel species and that IMCC44632^T^/IMCC44653 together represent a second novel species within the genus *Aliikangiella*. Genus-level affiliation was further supported by AAI values of 68.1–72.0% (Konstantinidis and Tiedje, 2007) and POCP values of 53.3–63.9% (Qin et al., 2014) relative to *Aliikangiella* species (Table S3). Concordantly, phylogenomic inference placed the three strains in a distinct, well-supported clade within the genus *Aliikangiella* (Fig. 2).

Genome-based metabolic reconstruction using KEGG module profiling revealed that the three strains share a heterotrophic, aerobic metabolic lifestyle (Table S4). In all genomes, the upper portion of the Embden–Meyerhof–Parnas pathway for glucose utilization (KEGG module ID: M00001) was incomplete, whereas the downstream glycolytic module for three-carbon intermediates (M00002) was complete. All strains encoded the non-oxidative pentose phosphate pathway (M00007) and PRPP biosynthesis (M00005) but lacked the oxidative pentose phosphate module (M00006), a configuration observed in other marine heterotrophic bacteria such as SAR11 and SAR86 (Dupont et al., 2012; Giovannoni et al., 2005). The Entner–Doudoroff pathway (M00008) was present only in IMCC44359^T^ and absent from IMCC44632^T^ and IMCC44653, indicating strain-level variation in carbohydrate catabolism. All three genomes encoded a complete TCA cycle (M00010 and M00011) and pyruvate oxidation (M00307), consistent with aerobic respiration. No canonical carbon fixation pathways (Calvin–Benson–Bassham cycle, reductive TCA cycle, Wood–Ljungdahl pathway, or 3-hydroxypropionate cycles) were detected, providing no genomic evidence for autotrophy or mixotrophy. Oxidative phosphorylation modules were complete in all strains, including NADH dehydrogenase (M00144), succinate dehydrogenase (M00149), cytochrome bc1 complex (M00151), cytochrome c oxidase (M00155), and F-type ATP synthase (M00157). Fatty acid biosynthesis and β-oxidation pathways were also considered complete (M00082, M00083, M00086, M00087). In addition, broad cofactor and vitamin biosynthesis potential was inferred (e.g., NAD, ubiquinone, coenzyme A, heme, biotin, riboflavin, siroheme, molybdenum cofactor, lipoic acid, and cobalamin). Collectively, these features are consistent with aerobic chemoorganotrophic marine bacteria that conserve energy via respiratory oxidation of organic substrates, in line with previously described members of *Aliikangiella*.

### Phenotypic characteristics

Transmission electron microscopy demonstrated that cells of all three strains were rod-shaped and motile by a single polar flagellum (Fig. S1), in agreement with the presence of flagellar assembly genes identified in their genomes. Cell dimensions were approximately 0.3–0.5 × 1.7–2.7 µm for IMCC44359^T^, 0.4–0.8 × 1.4–2.6 µm for IMCC44632^T^, and 0.6–0.8 × 3.1–3.8 µm for IMCC44653. The physiological and biochemical properties of strains IMCC44359^T^, IMCC44632^T^, and IMCC44653 are summarized in Tables 1 and S2, and the species protologues. The three IMCC isolates could be differentiated from previously described *Aliikangiella* species by distinct patterns of NaCl tolerance, starch hydrolysis, trypsin activity, and substrate oxidation profiles, supporting phenotypic separation.

Cellular fatty acid compositions of strains IMCC44359^T^, IMCC44632^T^, and IMCC44653, along with those of the type strains of the validly published *Aliikangiella* species, are presented in Table 2. Overall, the fatty acid profiles were consistent with affiliation to the genus *Aliikangiella*, being dominated by iso-C_15:0_ and summed feature 9 (iso-C_17:1_ ω9c and/or C_16:0_ 10-methyl). The dominance of iso-C_15:0_ and summed feature 9 has also been reported for “*A. maris*” GXAS 306^T^ (Li et al., 2025). The three novel strains shared several major fatty acids present at proportions greater than 10%, including iso-C_15:0_ (27.6%, 42.7%, and 42.9% in IMCC44359^T^, IMCC44632^T^, and IMCC44653, respectively) and summed feature 9 (19.2%, 26.9%, and 27.4%, respectively). Despite this overall similarity, clear quantitative differences were observed, particularly in the proportions of C_14:0_, C_16:0_, and iso-C_11:0_ 3-OH. These quantitative differences, together with variation in several minor unsaturated fatty acids, contributed to chemotaxonomic differentiation among the three strains and from previously described members of the genus.

All three strains possessed Q-8 as the sole respiratory quinone, consistent with the established chemotaxonomic characteristics of the genus *Aliikangiella*. The polar lipid profile of IMCC44359^T^ consisted of phosphatidylethanolamine (PE), phosphatidylglycerol (PG), diphosphatidylglycerol (DPG), one unidentified aminolipid (AL), and one unidentified lipid (Fig. S2). Strains IMCC44632^T^ and IMCC44653 exhibited comparable profiles comprising PE, PG, DPG, one unidentified aminophospholipid (APL), and three unidentified lipids. The shared presence of PE, PG, and DPG as major polar lipids corresponds with previously reported lipid patterns for members of the genus. Collectively, the fatty acid composition, quinone, and polar lipid characteristics provide coherent chemotaxonomic evidence supporting placement of these strains within the genus *Aliikangiella*.

### Taxonomic conclusion

Integrated evidence from 16S rRNA gene phylogeny, genome-scale relatedness indices, and physiological and chemotaxonomic characterization supported the placement of strains IMCC44359^T^, IMCC44632^T^, and IMCC44653 within the genus *Aliikangiella*. Strains IMCC44632^T^ and IMCC44653 exhibited genome relatedness values consistent with membership in a single species, whereas IMCC44359^T^ was clearly separated from these strains and from the two validly published *Aliikangiella* species by ANI and dDDH values well below the accepted species thresholds. These genomic distinctions, together with differentiating physiological and chemotaxonomic traits, support the recognition of two novel species within the genus *Aliikangiella*. Accordingly, we propose *Aliikangiella litoralis* sp. nov. (type strain IMCC44359^T^) and *Aliikangiella aequoris* sp. nov. (type strain IMCC44632^T^; reference strain IMCC44653).

### Description of *Aliikangiella litoralis* sp. nov.

*Aliikangiella litoralis* (li.to.ra′lis.; L. fem. adj. *litoralis*, coastal, of the shore)

Cells are Gram-stain-negative, strictly aerobic, motile rods, 0.3–0.5 µm in width and 1.7–2.7 µm in length. Colonies on marine agar are cream, circular, and convex. Growth occurs at 15–35℃ (optimum, 30℃), pH 6.0–9.0 (optimum, pH 8.0), and 1.0–4.0% (w/v) NaCl (optimum, 2.0%). Oxidase and catalase activities are positive. Hydrolyzes casein, Tween 20, and Tween 80, but not colloidal chitin, DNA, starch, and CM-cellulose. Esculin is not hydrolyzed. In API 20NE tests, nitrate reduction, and gelatinase are positive, whereas indole production, glucose fermentation, arginine dihydrolase, urease, and β-galactosidase activities are negative. Utilization of D-glucose, L-arabinose, D-mannose, D-mannitol, N-acetyl-glucosamine, D-maltose, potassium gluconate, capric acid, adipic acid, malic acid, trisodium citrate, and phenylacetic acid is negative. In API ZYM tests, alkaline phosphatase, esterase (C4), esterase lipase (C8), leucine arylamidase, valine arylamidase, α-chymotrypsin, acid phosphatase, and naphthol-AS-BI-phosphohydrolase activities are positive, but lipase (C14), cystine arylamidase, trypsin, α-galactosidase, β-galactosidase, β-glucuronidase, α-glucosidase, β-glucosidase, N-acetyl-β-glucosaminidase, α-mannosidase, and α-fucosidase activities are negative. In the GEN III MicroPlate, oxidizes L-histidine, L-serine, glucuronamide, L-malic acid, and acetoacetic acid. Predominant fatty acids are C_14:0_, C_16:0_, iso-C_15:0_, iso-C_11:0_ 3-OH, and summed feature 9 (iso-C_17:1_ ω9c and/or C_16:0_ 10-methyl). The major respiratory quinone is Q-8. Major polar lipids are phosphatidylethanolamine, phosphatidylglycerol, diphosphatidylglycerol, one unidentified aminolipid, and one unidentified lipid.

The type strain is IMCC44359^T^ (= KCTC 18089^T^ = JCM 37879^T^ = HNIBRBA19635^T^) isolated from surface seawater off Jangbong Island, Republic of Korea. The genome size of the type strain is 5,951,045 bp and the DNA G + C content of 36.5%. The GenBank accession numbers for the 16S rRNA gene sequence and genome sequence are PX239437 and CP199292, respectively.

### Description of *Aliikangiella aequoris* sp. nov.

*Aliikangiella aequoris* (ae'quo.ris.; L. gen. n. *aequoris*, of the sea)

Cells are Gram-stain-negative, strictly aerobic, motile rods, 0.4–0.8 µm in width and 1.4–3.8 µm in length. Colonies on marine agar are cream, circular, and convex. Growth occurs at 10–40℃ (optimum, 30℃), pH 6.0–9.0 (optimum, pH 7.0–8.0), and 0.5–7.5% (w/v) NaCl (optimum, 2.0–3.0%). Oxidase activity is positive and catalase activity is negative. Hydrolyzes casein, Tween 20, and Tween 80, but not colloidal chitin, DNA, starch, and CM-cellulose. Esculin is not hydrolyzed. In API 20NE tests, gelatinase is positive, whereas nitrate reduction, indole production, glucose fermentation, arginine dihydrolase, urease, and β-galactosidase are negative. Utilization of D-glucose, L-arabinose, D-mannose, D-mannitol, N-acetyl-glucosamine, D-maltose, potassium gluconate, capric acid, adipic acid, malic acid, trisodium citrate, and phenylacetic acid is negative. In API ZYM tests, alkaline phosphatase, esterase (C4), esterase lipase (C8), leucine arylamidase, and naphthol-AS-BI-phosphohydrolase activities are positive, but lipase (C14), trypsin, α-chymotrypsin, α-galactosidase, β-galactosidase, β-glucuronidase, α-glucosidase, β-glucosidase, N-acetyl-β-glucosaminidase, α-mannosidase, and α-fucosidase activities are negative. Activities of valine arylamidase, cystine arylamidase, and acid phosphatase are variable. In the GEN III MicroPlate, oxidizes glucuronamide and L-malic acid. Oxidation of D-malic acid and bromo-succinic acid is variable. Predominant fatty acids are iso-C_15:0_ and summed feature 9 (iso-C_17:1_ ω9c and/or C_16:0_ 10-methyl). The major respiratory quinone is Q-8. Major polar lipids are phosphatidylethanolamine, phosphatidylglycerol, diphosphatidylglycerol, one unidentified aminophospholipid, and three unidentified lipids. The genome size is approximately 3.75 Mbp and the DNA G + C content is 40.1–40.2%.

The type strain is IMCC44632^T^ (= KCTC 18090^T^ = JCM 37880^T^ = HNIBRBA19636^T^), isolated from coastal seawater off Jangbong Island, Republic of Korea. The GenBank accession numbers for the 16S rRNA gene sequence and genome sequence are PX239438 and JBSKFV000000000, respectively.

## Figures and Tables

**Table 1.** Differential phenotypic characteristics of strains IMCC44359^T^, IMCC44632^T^, and IMCC44653 and the type strains of *Aliikangiella* species Strains: 1, IMCC44359^T^; 2, IMCC44632^T^; 3, IMCC44653; 4, *A. marina* KCTC 42667^T^; 5, *A. coralliicola* KCTC 72442^T^: 6, “*A. maris*” GXAS 306^T^. Data are from this study unless otherwise indicated. All strains are positive for degradation of casein, Tween 20, and Tween 80, and enzyme activities for gelatinase, alkaline phosphatase, esterase (C4), esterase lipase (C8), leucine arylamidase, and naphthol-AS-BI-phosphohydrolase. Detailed carbon source oxidation pattern is summarized in Table S2. +, Positive; ‒, negative; v, variable; nd, not determined.

Characteristics	1	2	3	4	5	6^c^
Colony color	cream	cream	cream	pale yellow–green	pale-yellow	pale-yellow
Growth at						
Temperature range (optimum, ºC)	10–35 (30)	10–40 (30)	10–40 (30)	15–37 (30)	15–40 (25–30)	15–37 (28–30)
pH range (optimum)	6–9 (8)	6–9 (8)	6–9 (7)	6–9 (7–8)	7–8 (7)	5.5–10.5 (6–7)
NaCl range (optimum, %)	1–4 (2)	0.5–7.5 (3)	0.5–7.5 (2)	1–5 (2–3)	0–10 (1–3)	0–4 (2–3)
Oxidase	+	+	+	+	–	–
Catalase	+	–	–	+	+	+
Hydrolysis of:						
Starch	–	–	–	+	+	–
Esculin	–	–	–	+^a^	+	nd
API 20NE						
Nitrate reduction	+	–	–	–^a^	+	nd
*β*-Galactosidase	–	–	–	–^a^	+	nd
API ZYM						
Lipase (C14)	–	–	–	+	–^b^	+
Valine arylamidase, Acid phosphatase	+	–	+	+	+^b^	v
Cystine arylamidase	–	–	+	+	+^b^	+
Trypsin	–	–	–	+	+^b^	+
*α*-Chymotrypsin	+	–	–	+	+^b^	+
GEN III						
Gentiobiose, Sucrose, N-Acetyl-D-Glucosamine, N-Acetyl-*β*-D-Mannosamine, D-Fucose, L-Fucose	–	–	–	–	+	nd
L-Histidine, L-Serine	+	–	–	–	–	nd
D-Malic acid	–	+	–	+	+	nd
Bromo-succinic acid	–	+	–	+	–	nd
Acetoacetic acid	+	–	–	–	+	nd

a Data from Wang et al. (2015).

b Data from Wang et al. (2020).

c Data from Li et al. (2025).

**Table 2.** Cellular fatty acid compositions (%) of strains IMCC44359^T^, IMCC44632^T^, and IMCC44653 and the type strains of validly published *Aliikangiella* species Strains: 1, IMCC44359^T^; 2, IMCC44632^T^; 3, IMCC44653; 4, *A. marina* KCTC 42667^T^; 5, *A. coralliicola* KCTC 72442^T^; All data are from this study. All strains were cultured under identical conditions. Fatty acids representing < 1.0% of the total in all species were omitted. ‒, Not detected; Tr, traces (< 1.0%). Major fatty acids (> 10%) are shown in bold.

Fatty acid (%)	1	2	3	4	5
Saturated					
C_14:0_	10.3	2.1	1.9	Tr	1.0
C_16:0_	16.0	1.7	1.6	4.9	2.7
C_18:0_	Tr	–	–	1.5	Tr
Branched					
iso-C_13:0_	Tr	1.2	1.1	Tr	–
iso-C_14:0_	Tr	1.3	1.4	1.7	2.4
iso-C_15:0_	27.6	42.7	42.9	34.6	29.7
iso-C_16:0_	2.3	3.8	4.1	9.9	8.1
iso-C_17:0_	2.5	1.7	1.6	7.3	12.6
Hydroxy					
iso-C_10:0_ 3-OH	2.3	Tr	–	Tr	Tr
iso-C_11:0_ 3-OH	10.4	9.0	8.5	3.0	4.0
Unsaturated					
iso-F C_15:1_	Tr	3.3	3.0	Tr	1.3
C_16:1_ iso H	–	Tr	Tr	1.6	1.2
Summed Feature					
Summed feature 3^*^	5.1	5.4	5.1	5.3	9.6
Summed feature 8^*^	Tr	–	–	–	2.4
Summed feature 9^*^	19.2	26.9	27.4	25.5	20.1

* Summed feature 3 consisted of C_16:1_ ω7c and/or C_16:1_ ω6c, summed feature 8 contained C_18:1_ ω7c and/or C_18:1_ ω6c and summed feature 9 consisted of iso-C_17:1_ ω9c and/or C_16:0_ 10-methyl.

## Supplementary Information

Table S1. General genomic features of strains IMCC44359^T^, IMCC44632^T^, and IMCC44653 and the type strains of *Aliikangiella* species Strains: 1, IMCC44359^T^; 2, IMCC44632^T^; 3, IMCC44653; 4, *A. marina* GYP-15^T^; 5, *A. coralliicola* M105^T^; 6, “*A. maris*” GXAS 306^T^.

Table S2. Carbon source oxidation patterns determined using the GENⅢ MicroPlate system for strains IMCC44359^T^, IMCC44632^T^, IMCC44653, *A. marina* KCTC 42667^T^, and *A. coralliicola* KCTC 72442^T^ Strains: 1, IMCC44359^T^; 2, IMCC44632^T^; 3, IMCC44653; 4, *A. marina* KCTC 42667^T^; 5, *A. coralliicola* KCTC 72442^T^. All data are from this study. +, positive; ‒, negative.

Table S3. Average amino acid identity (AAI; lower triangle) and percentage of conserved proteins (POCP; upper triangle) among strains IMCC44359^T^, IMCC44632^T^, IMCC44653, and related *Aliikangiella* species. Values represent percentages. Strains: 1, IMCC44359^T^; 2, IMCC44632^T^; 3, IMCC44653; 4, *A. marina* GYP-15^T^; 5, *A. coralliicola* M105^T^; 6, *A. maris* GXAS 306^T^.

Table S4. KEGG module profiles of strains IMCC44359^T^, IMCC44632^T^, IMCC44653, *Aliikangiella marina* GYP-15^T^, *Aliikangiella coralliicola* M105^T^, and *Aliikangiella maris* GXAS 306^T^

Fig. S1. Transmission electron micrographs of cells of strains IMCC44359^T^ (A), IMCC44632^T^ (B), and IMCC44653(C) after negative staining with 1% (w/v) uranyl acetate. Cells were grown on marine agar at 25°C for 5 days. Bar, 0.5 μm.

Fig. S2. Two-dimensional thin-layer chromatogram of polar lipids of strains IMCC44359^T^ (A), IMCC44632^T^ (B), and IMCC44653 (C). PE, phosphatidylethanolamine; PG, phosphatidylglycerol; DPG, diphosphatidylglycerol; AL, unidentified aminolipid; APL, unidentified aminophospholipid; L, unidentified lipid.
