# Supplementary material for: Aliikangiella litoralis sp. nov. and Aliikangiella aequoris sp. nov., isolated from coastal seawaters of the Yellow Sea
Source: J Microbiol. 2026 May 19;64(5):e2602008. doi: 10.71150/jm.2602008 (PMC13576998; doi:10.71150/jm.2602008)

Fig. S1. Transmission electron micrographs of cells of strains IMCC44359^{T} (A), IMCC44632^{T} (B), and IMCC44653(C) after negative staining with 1% (w/v) uranyl acetate. Cells were grown on marine agar at 25°C for 5 days. Bar, 0.5 μm.

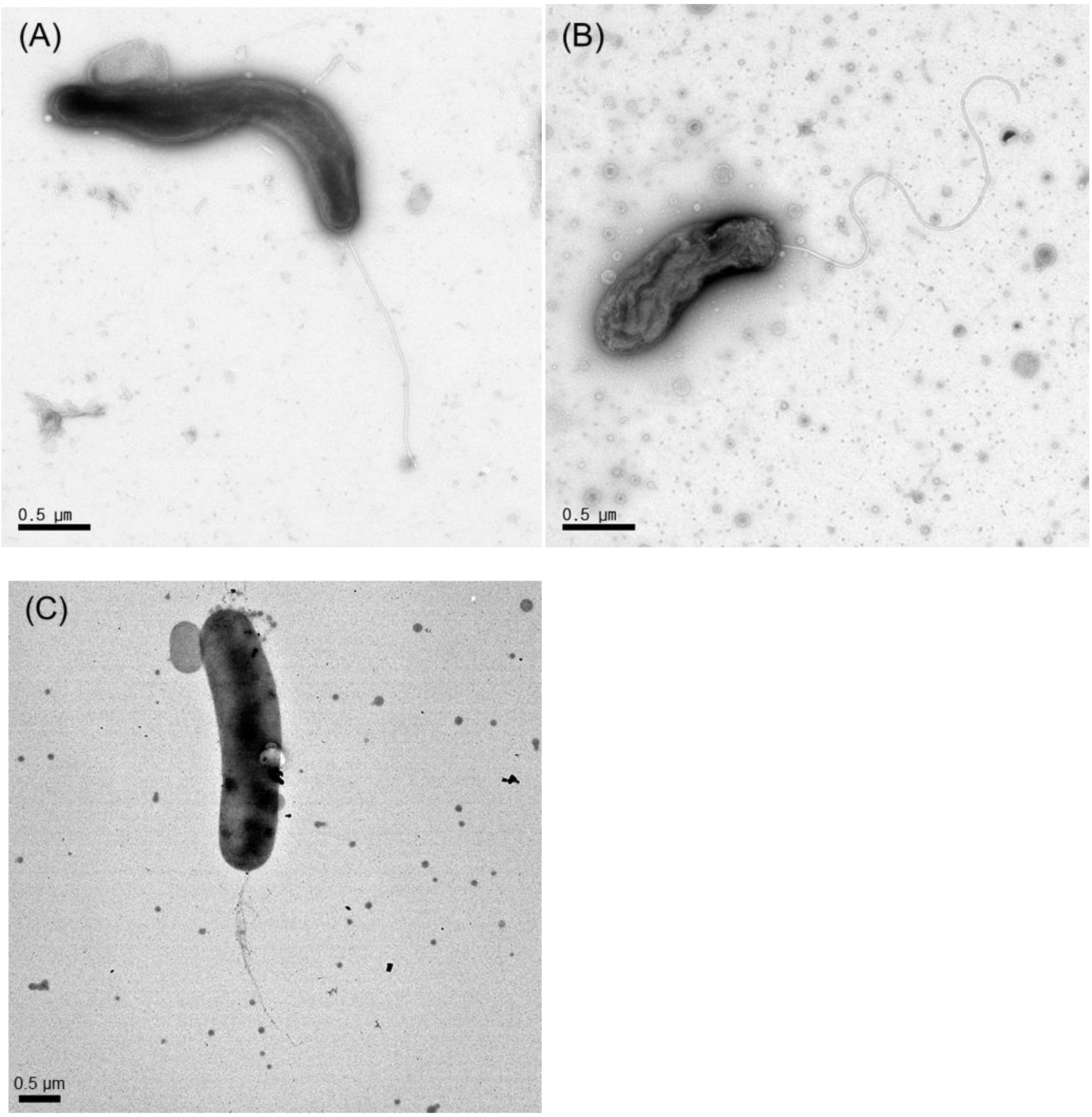

Supplement: Supplementary file 1 [file jm-2602008-Supplementary-Fig-S1.pdf]
