# Supplementary material for: Aliikangiella litoralis sp. nov. and Aliikangiella aequoris sp. nov., isolated from coastal seawaters of the Yellow Sea
Source: J Microbiol. 2026 May 19;64(5):e2602008. doi: 10.71150/jm.2602008 (PMC13576998; doi:10.71150/jm.2602008)

Fig. S2. Two-dimensional thin-layer chromatogram of polar lipids of strains IMCC44359^{T} (A), IMCC44632^{T} (B), and IMCC44653 (C). PE, phosphatidylethanolamine; PG, phosphatidylglycerol; DPG, diphosphatidylglycerol; AL, unidentified aminolipid; APL, unidentified aminophospholipid; L, unidentified lipid.

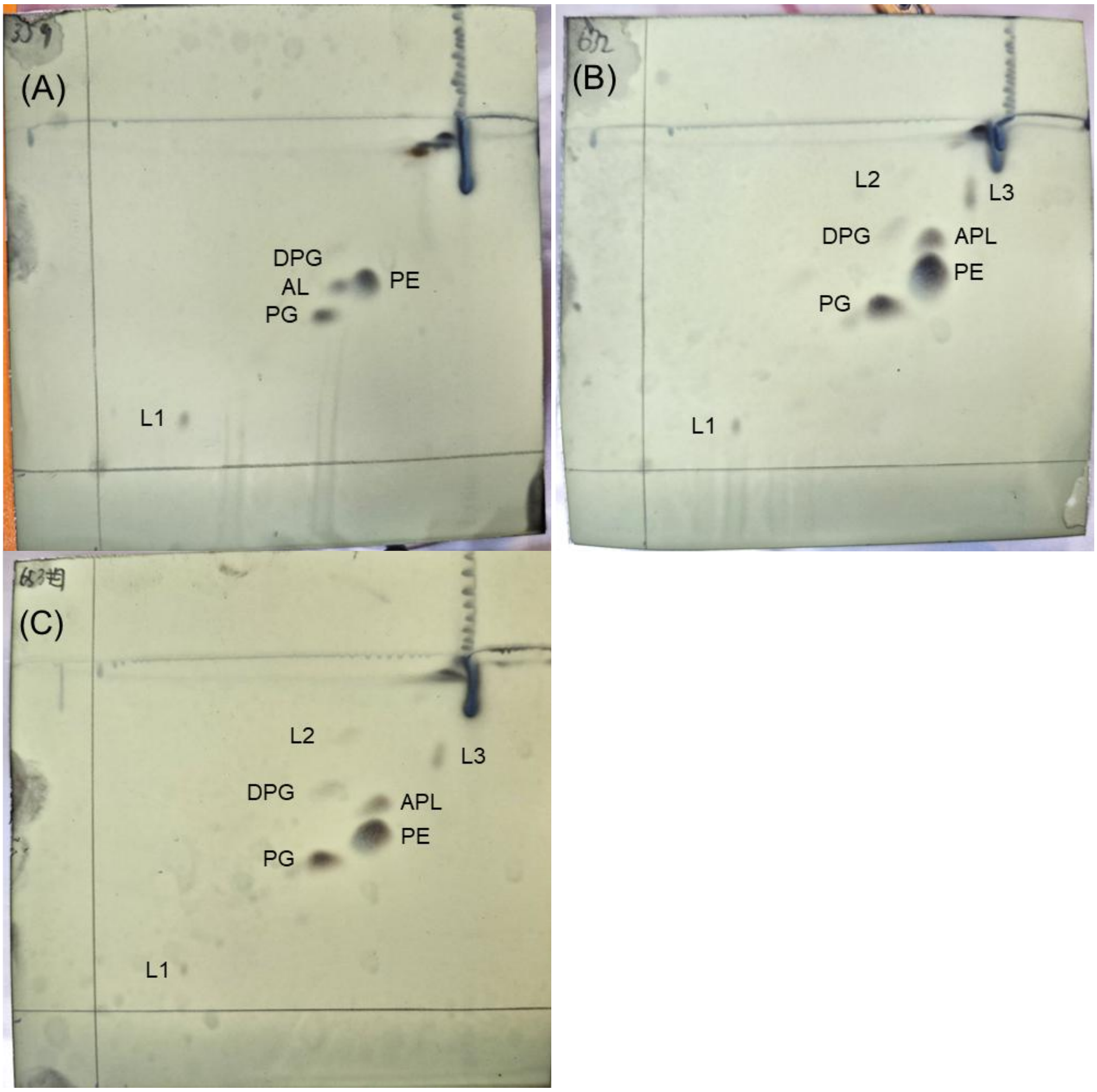

Supplement: Supplementary file 2 [file jm-2602008-Supplementary-Fig-S2.pdf]
