# Supplementary material for: Aliikangiella litoralis sp. nov. and Aliikangiella aequoris sp. nov., isolated from coastal seawaters of the Yellow Sea
Source: J Microbiol. 2026 May 19;64(5):e2602008. doi: 10.71150/jm.2602008 (PMC13576998; doi:10.71150/jm.2602008)
Supplement: Supplementary file 3 [file jm-2602008-Supplementary-Table-S1.pdf]

Table S1. General genomic features of strains IMCC44359^T, IMCC44632^T, and IMCC44653 and the type strains of *Aliikangiella* species

Strains: 1, IMCC44359^T; 2, IMCC44632^T; 3, IMCC44653; 4, *A. marina* GYP-15^T; 5, *A. coralliicola* M105^T; 6, "*A. maris*" GXAS 306^T.

Strain	1	2	3	4	5	**6**
GenBank accession number	CP199292	JBSKFV000000000	JBSKFW000000000	VIKR00000000*	VIKS00000000*	JBFDAH000000000*
Size (bp)	5,951,045	3,753,390	3,750,766	5,336,331	7,044,713	5,379,662
Number of contigs	1	1	1	8	22	118
G + C content (%)	36.5	40.1	40.2	41.9	41.5	38.5
Number of tRNA	50	43	43	58	41	49
Number of rRNA	6	9	9	5	5	3
Protein coding sequence	4,928	3,229	3,231	4,537	5,428	4,457

*Genomes of *A. marina* GYP-15^T, *A. coralliicola* M105^T, and "*A. maris*" GXAS 306^T were downloaded from the NCBI GenBank database and annotated in this study for comparative genomic analysis.
