# Supplementary material for: Aliikangiella litoralis sp. nov. and Aliikangiella aequoris sp. nov., isolated from coastal seawaters of the Yellow Sea
Source: J Microbiol. 2026 May 19;64(5):e2602008. doi: 10.71150/jm.2602008 (PMC13576998; doi:10.71150/jm.2602008)
Supplement: Supplementary file 4 [file jm-2602008-Supplementary-Table-S2.pdf]

Table S2. Carbon source oxidation patterns determined using the GENIII MicroPlate system for strains IMCC44359^T, IMCC44632^T, IMCC44653, *A. marina* KCTC 42667^T, and *A. coralliicola* KCTC 72442^T

Strains: 1, IMCC44359^T; 2, IMCC44632^T; 3, IMCC44653; 4, *A. marina* KCTC 42667^T; 5, *A. coralliicola* KCTC 72442^T. All data are from this study. +, positive; –, negative.

Oxidation of carbons	1	2	3	4	5
Dextrin	–	–	–	–	–
D-Maltose	–	–	–	–	–
D-Trehalose	–	–	–	–	–
D-Cellobiose	–	–	–	–	–
Gentiobiose	–	–	–	–	+
Sucrose	–	–	–	–	+
D-Turanose	–	–	–	–	–
Stachyose	–	–	–	–	–
D-Raffinose	–	–	–	–	–
α-D-Lactose	–	–	–	–	–
D-Melibiose	–	–	–	–	–
β-Methyl-D-glucoside	–	–	–	–	–
D-Salicin	–	–	–	–	–
N-Acetyl-D-glucosamine	–	–	–	–	+
N-Acetyl-*β*-D-mannosamine	–	–	–	–	+
N-Acetyl-D-galactosamine	–	–	–	–	–
N-Acetyl neuraminic acid	–	–	–	–	–
α-D-Glucose	–	–	–	–	–
D-Mannose	–	–	–	–	–
D-Fructose	–	–	–	–	–
D-Galactose	–	–	–	–	–
3-Methyl-D-glucose	–	–	–	–	–
D-Fucose	–	–	–	–	+
L-Fucose	–	–	–	–	+
L-Rhamnose	–	–	–	–	–
Inosine	–	–	–	–	–
D-Sorbitol	–	–	–	–	–
D-Mannitol	–	–	–	–	–
D-Arabitol	–	–	–	–	–
myo-Inositol	–	–	–	–	–
Glycerol	–	–	–	–	–

D-Glucose-6-phosphate	–	–	–	–	–
D-Fructose-6-phosphate	–	–	–	–	–
D-Aspartic acid	–	–	–	–	–
D-Serine	–	–	–	–	–
Gelatin	–	–	–	–	–
Glycyl-L-proline	–	–	–	–	–
L-Alanine	–	–	–	–	–
L-Arginine	–	–	–	–	–
L-Aspartic acid	–	–	–	–	–
L-Glutamic acid	–	–	–	–	–
L-Histidine	+	–	–	–	–
L-Pyroglutamic acid	–	–	–	–	–
L-Serine	+	–	–	–	–
Pectin	–	–	–	–	–
D-Galacturonic acid	–	–	–	–	–
L-Galactonic acid lactone	–	–	–	–	–
D-Gluconic acid	–	–	–	–	–
D-Glucuronic acid	–	–	–	–	–
Glucuronamide	+	+	+	+	+
Mucic acid	–	–	–	–	–
Quinic acid	–	–	–	–	–
D-Saccharic acid	–	–	–	–	–
p-Hydroxy phenylacetic acid	–	–	–	–	–
Methyl pyruvate	–	–	–	–	–
D-Lactic acid methyl ester	–	–	–	–	–
L-Lactic acid	–	–	–	–	–
Citric acid	–	–	–	–	–
α-Ketoglutaric acid	–	–	–	–	–
D-Malic acid	–	+	–	+	+
L-Malic acid	+	+	+	+	+
Bromo-succinic acid	–	+	–	+	–
Tween 40	–	–	–	–	–
γ-Amino-butyric acid	–	–	–	–	–
α-Hydroxybutyric acid	–	–	–	–	–
β-Hydroxy-D,L-butyric acid	–	–	–	–	–
α-Ketobutyric acid	–	–	–	–	–
Acetoacetic acid	+	–	–	-	+
Propionic acid	–	–	–	–	–
Acetic acid	–	–	–	–	–
Formic acid	–	–	–	–	–
