# Supplementary material for: Aliikangiella litoralis sp. nov. and Aliikangiella aequoris sp. nov., isolated from coastal seawaters of the Yellow Sea
Source: J Microbiol. 2026 May 19;64(5):e2602008. doi: 10.71150/jm.2602008 (PMC13576998; doi:10.71150/jm.2602008)
Supplement: Supplementary file 5 [file jm-2602008-Supplementary-Table-S3.pdf]

Table S3. Average amino acid identity (AAI; lower triangle) and percentage of conserved proteins (POCP; upper triangle) among strains IMCC44359^T, IMCC44632^T, IMCC44653, and related *Aliikangiella* species. Values represent percentages.

Strains: 1, IMCC44359^T; 2, IMCC44632^T; 3, IMCC44653; 4, *A. marina* GYP-15^T; 5, *A. coralliicola* M105^T; 6, *A. maris* GXAS 306^T.

Strain	1	2	3	4	5	6
1	–	57.4	57.4	54.5	56.6	63.9
2	69.2	–	94.1	60.4	53.3	59.2
3	69.0	99.3	–	60.4	53.5	59.1
4	69.5	68.2	68.1	–	55.9	54.4
5	69.8	68.6	68.6	69.4	–	52.9
6	72.0	68.0	68.0	68.4	68.3	–

*Genomes of *A. marina* GYP-15^T, *A. coralliicola* M105^T, and "*A. maris*" GXAS 306^T were downloaded from the NCBI GenBank database and annotated in this study for comparative genomic analysis.
